# Supplementary figures and images for: GRID-independent molecular descriptor analysis and molecular docking studies to mimic the binding hypothesis of γ-aminobutyric acid transporter 1 (GAT1) inhibitors
Source: PeerJ. 2019 Jan 31;7:e6283. doi: 10.7717/peerj.6283 (PMC6360079; doi:10.7717/peerj.6283)

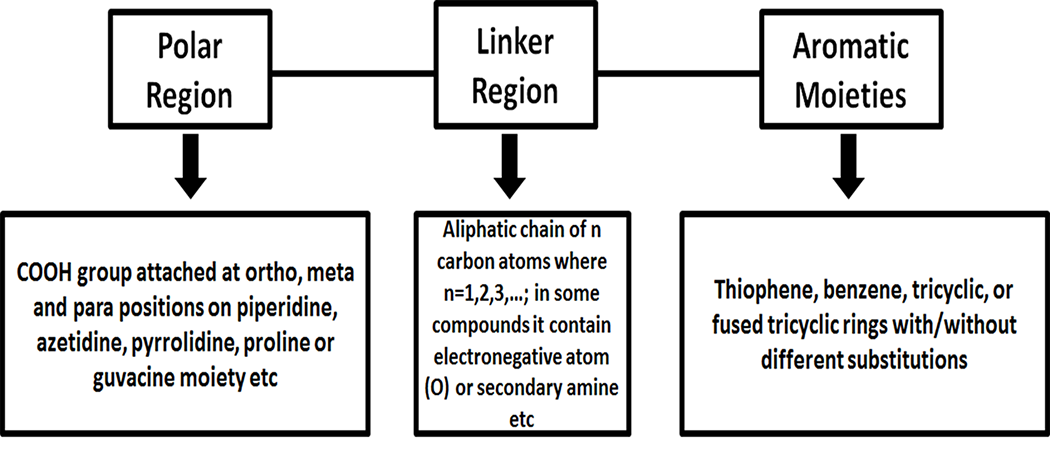

Supplement: Supplemental Information 1 [file peerj-07-6283-s001.png]

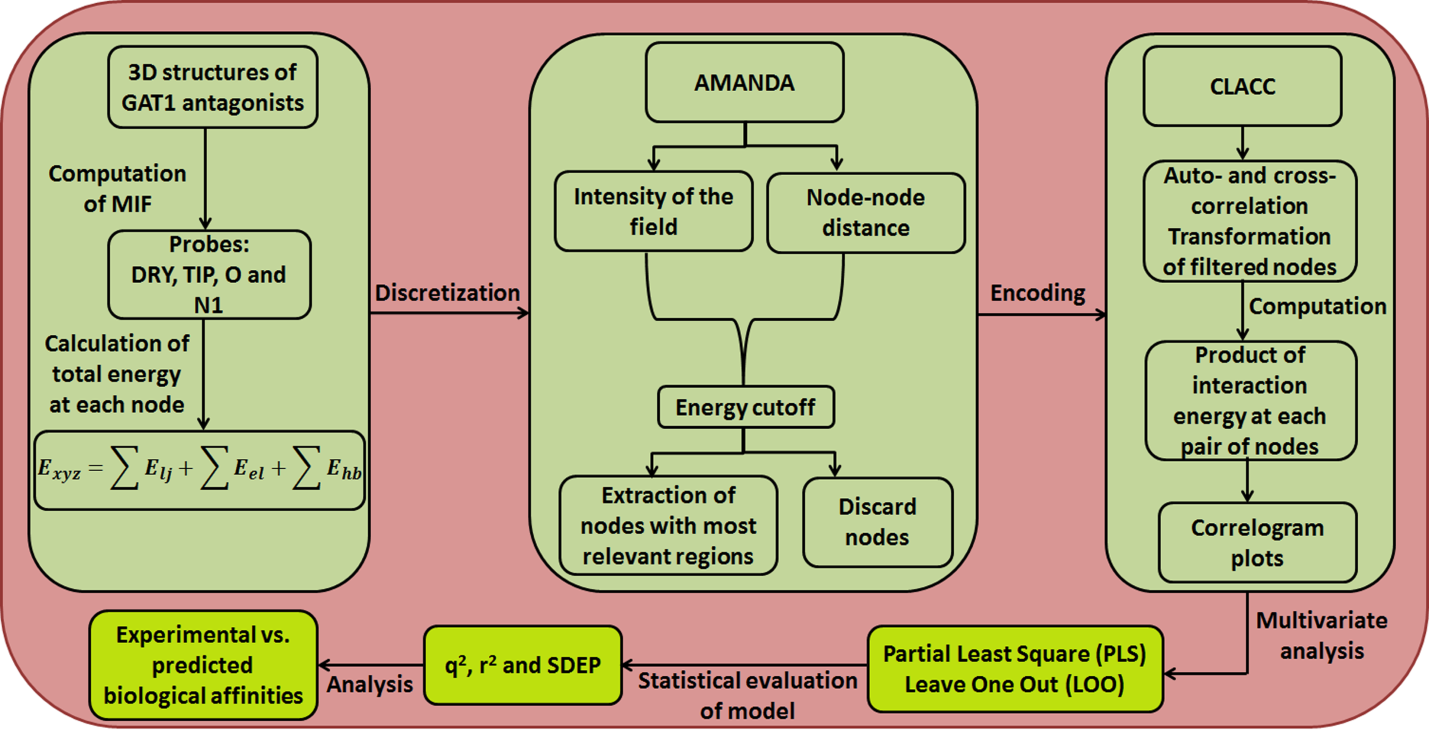

Supplement: Supplemental Information 2 [file peerj-07-6283-s002.png]

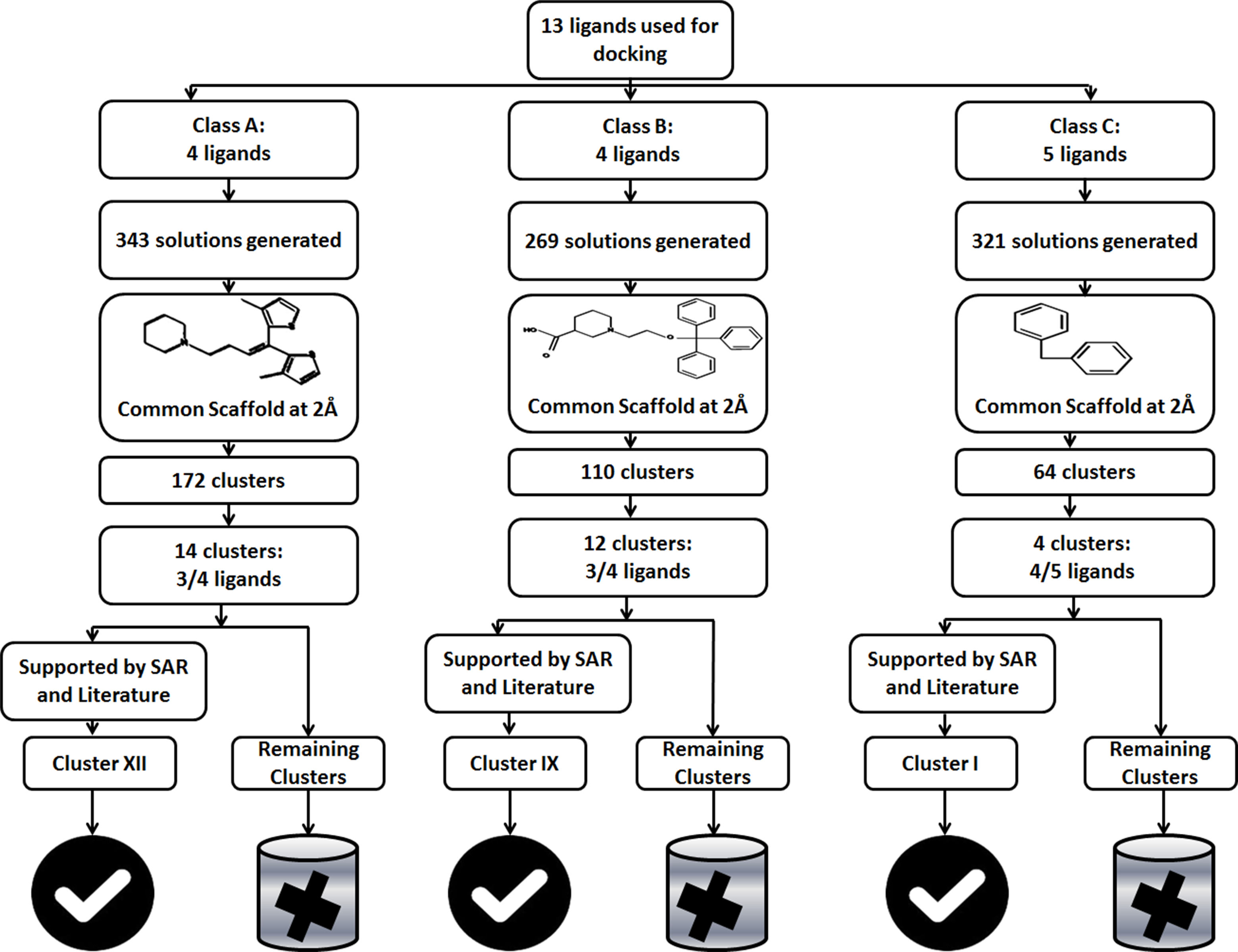

Supplement: Supplemental Information 3 [file peerj-07-6283-s003.png]

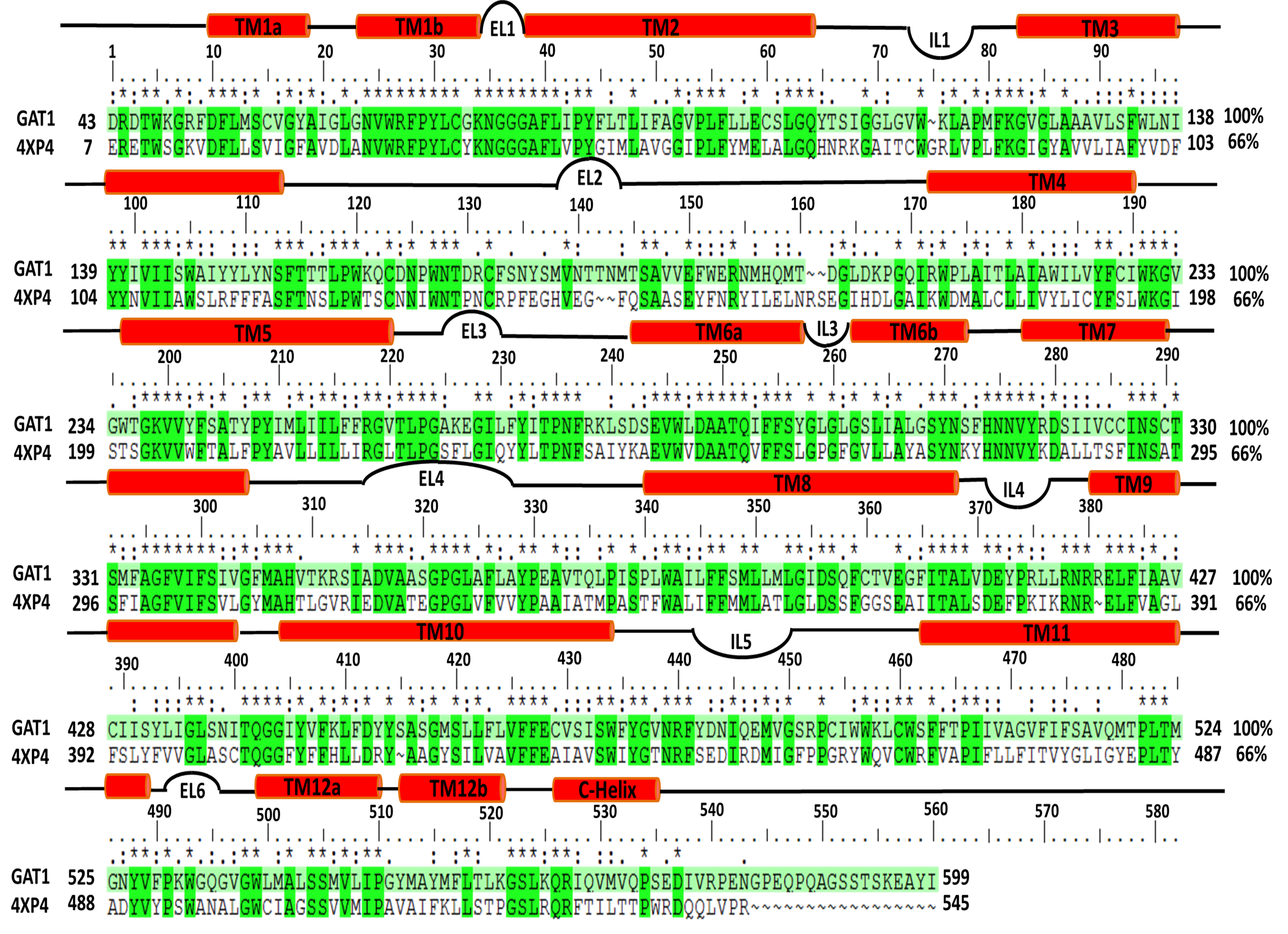

Supplement: Supplemental Information 4 [file peerj-07-6283-s004.png]

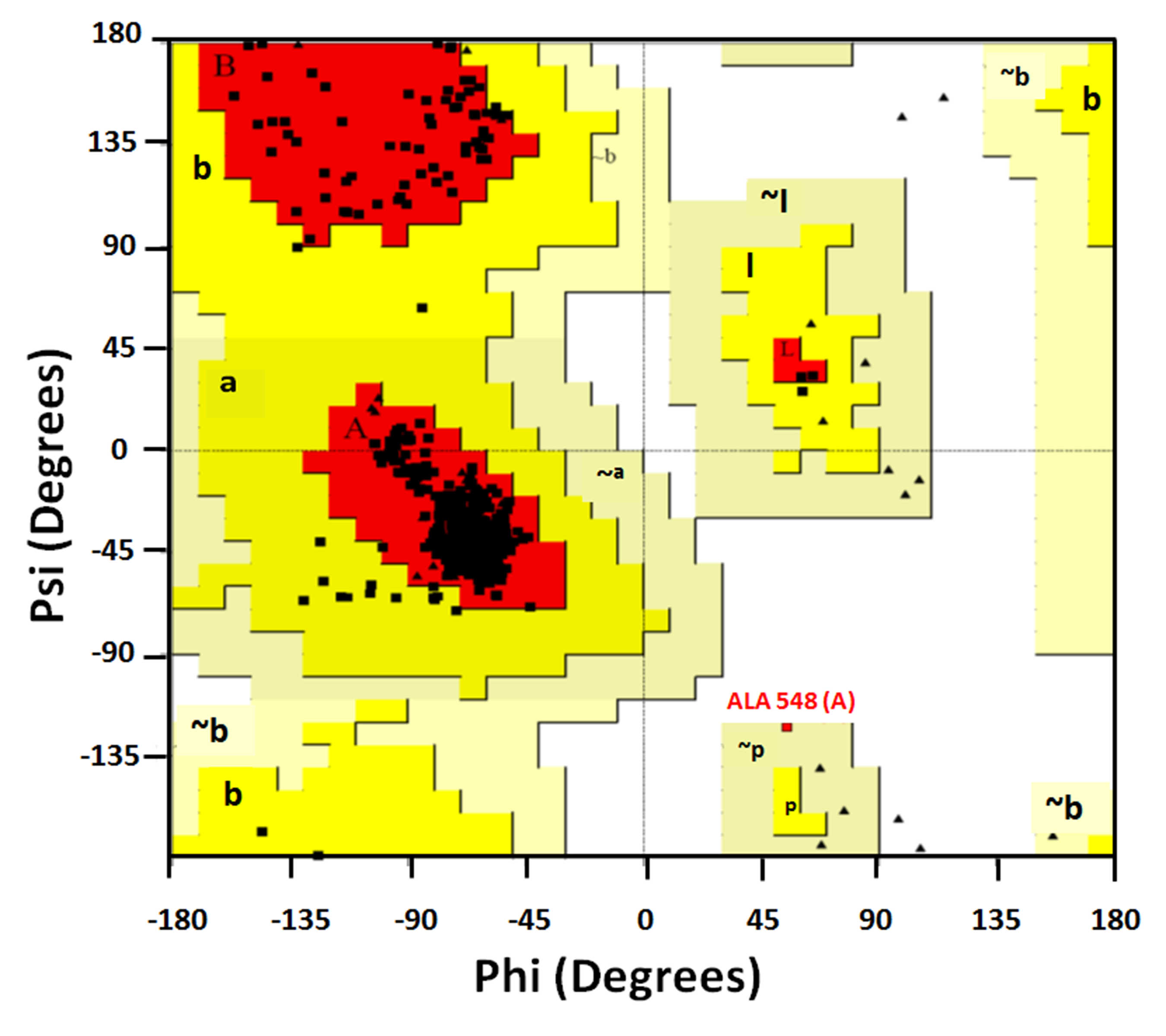

Supplement: Supplemental Information 5 — The most favored regions are marked as A, B and L whereas additionally allowed regions are indicated by a, b, l and p. Proline and non-glycine residues are marked as black squares. Filled tringles represent glycine residues. [file peerj-07-6283-s005.png]

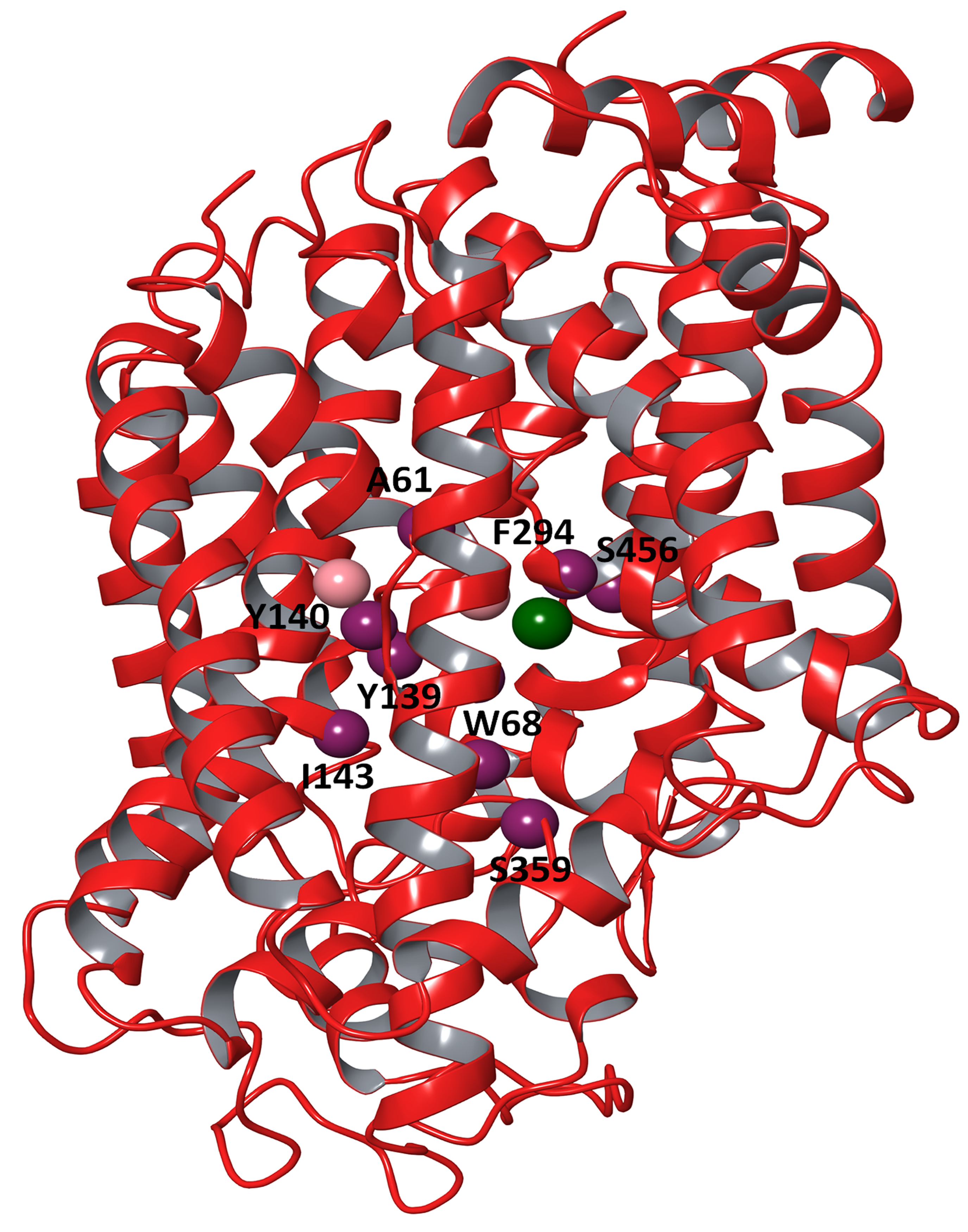

Supplement: Supplemental Information 6 — N-terminus and C-terminus residues are removed. Na+ ions are represented by blue spheres and Cl− ion by green sphere. [file peerj-07-6283-s006.png]

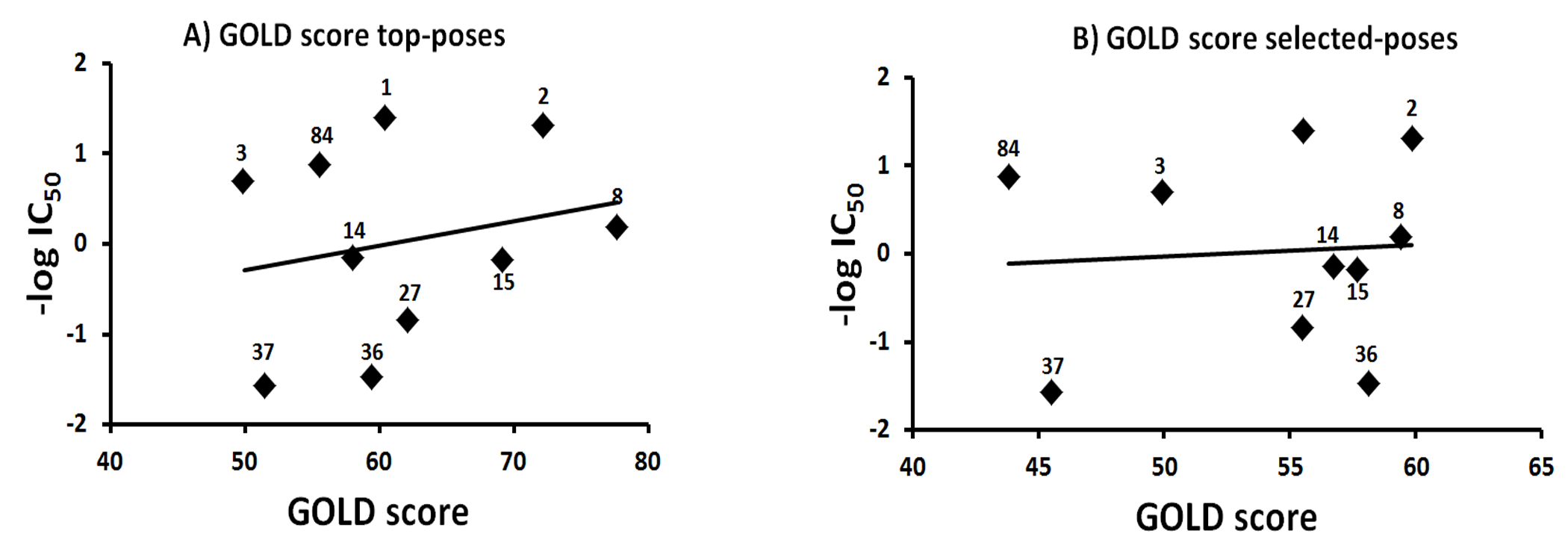

Supplement: Supplemental Information 7 [file peerj-07-6283-s007.png]

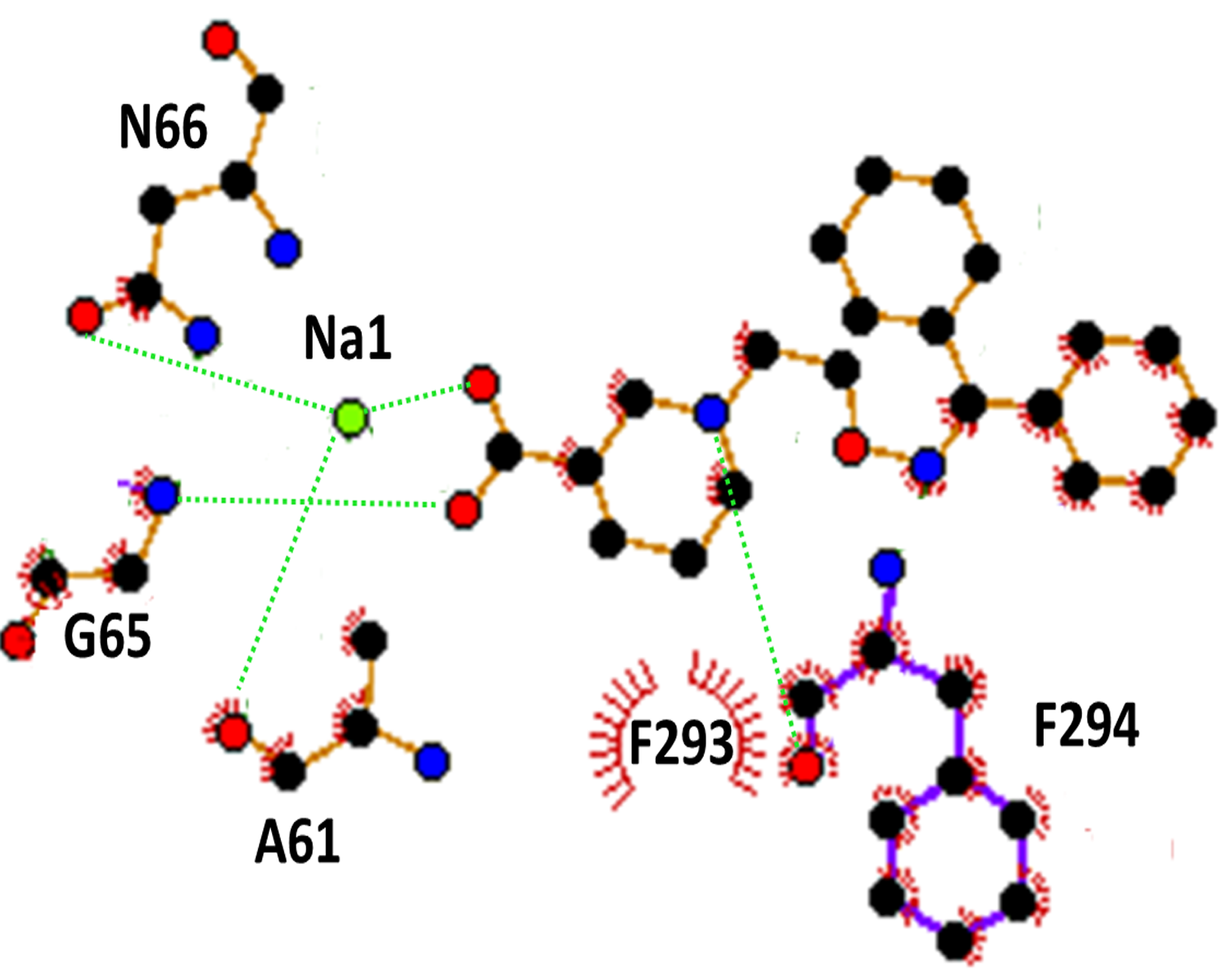

Supplement: Supplemental Information 8 [file peerj-07-6283-s008.png]

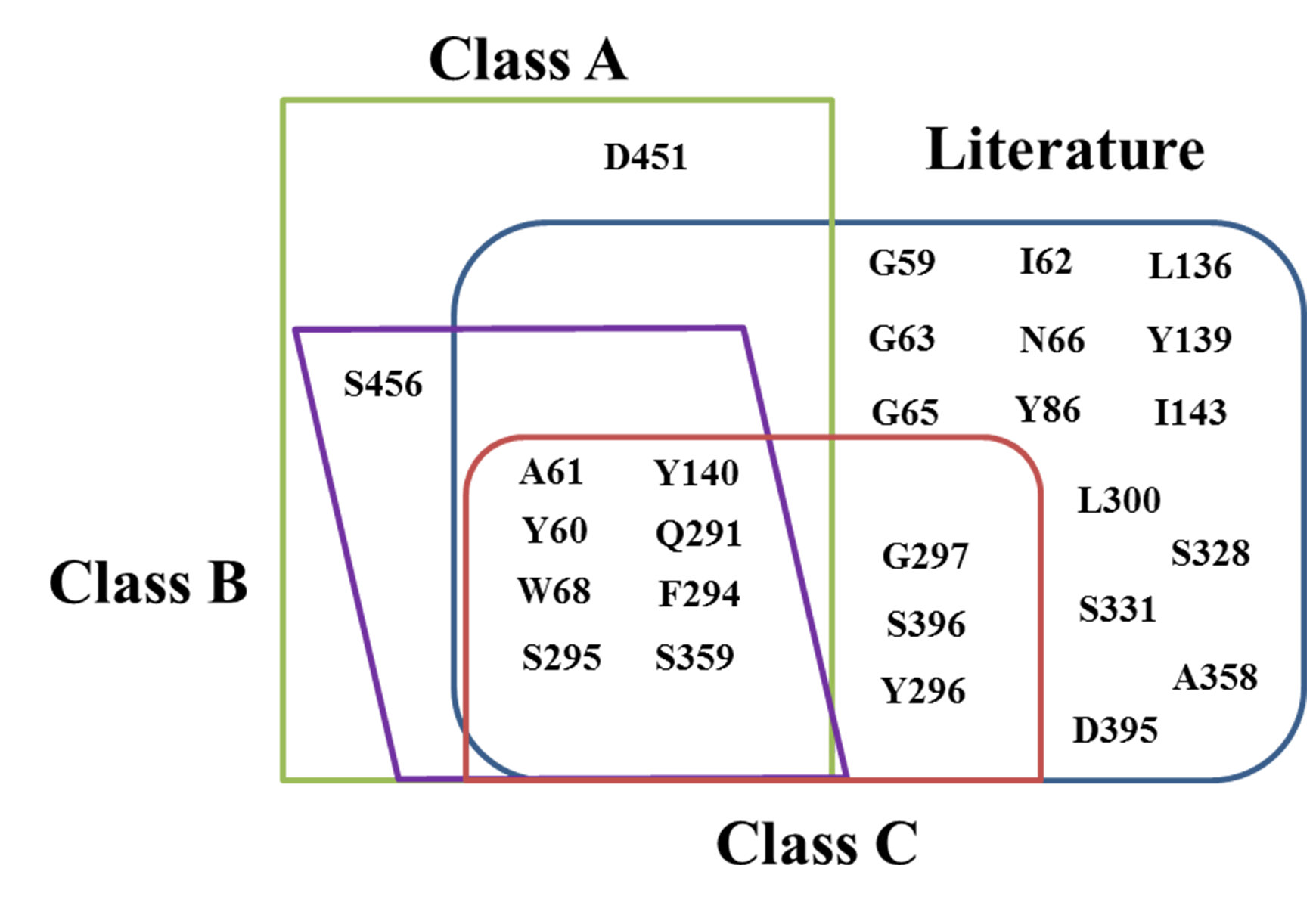

Supplement: Supplemental Information 9 [file peerj-07-6283-s009.png]
