# Supplementary material for: GRID-independent molecular descriptor analysis and molecular docking studies to mimic the binding hypothesis of γ-aminobutyric acid transporter 1 (GAT1) inhibitors
Source: PeerJ. 2019 Jan 31;7:e6283. doi: 10.7717/peerj.6283 (PMC6360079; doi:10.7717/peerj.6283)
Supplement: Supplemental Information 11 [file peerj-07-6283-s011.docx]

**S2 Table:** Statistical parameters of PLS models developed by using multiple conformational sets at pH 7.4.

| **Sr. No.** | **Conformational set** | **Full set of variables** | | | **FFD1** | | | **FFD2** | | |
| --- | --- | --- | --- | --- | --- | --- | --- | --- | --- | --- |
|  |  | **q^2^** | **r^2^** | **SDEP** | **q^2^** | **r^2^** | **SDEP** | **q^2^** | **r^2^** | **SDEP** |
|  | Extended 3D conformations | -0.13 | 0.36 | 0.74 | 0.02 | 0.36 | 0.69 | 0.10 | 0.35 | 0.66 |
| 2. | Energy minimized conformations | 0.11 | 0.34 | 0.66 | 0.26 | 0.41 | 0.60 | 0.34 | 0.47 | 0.57 |
| 3. | Induced fit docking conformations | 0.00 | 0.20 | 0.70 | 0.19 | 0.48 | 0.63 | 0.32 | 0.55 | 0.58 |
| 4. | Flexible alignment by pharmacophore mapping approach | 0.32 | 0.80 | 0.58 | 0.50 | 0.75 | 0.50 | **0.59** | **0.75** | **0.44** |
