# Supplementary material for: GRID-independent molecular descriptor analysis and molecular docking studies to mimic the binding hypothesis of γ-aminobutyric acid transporter 1 (GAT1) inhibitors
Source: PeerJ. 2019 Jan 31;7:e6283. doi: 10.7717/peerj.6283 (PMC6360079; doi:10.7717/peerj.6283)
Supplement: Supplemental Information 12 [file peerj-07-6283-s012.docx]

**S3 Table:** Test dataset, experimental vs. predicted -log IC_50_ and their respective residual values obtained via GRIND

| **Compound ID** | **Experimental -log IC_50_** | **Predicted -log IC_50_** | **Residual value** |
| --- | --- | --- | --- |
| 84 | 0.88 | -0.01 | 0.89 |
| 85 | -0.51 | -0.46 | -0.04 |
| 86 | -0.71 | -0.56 | -0.14 |
| 87 | -0.49 | -0.68 | 0.19 |
| 88 | -0.84 | 0.60 | -0.24 |
| 89 | -0.60 | -0.66 | 0.05 |
| 90 | -0.47 | -0.24 | -0.22 |
| 91 | 0.40 | -0.07 | 0.47 |
| 92 | -0.48 | -0.11 | -0.37 |
| 93 | 0.05 | -0.15 | 0.21 |
| 94 | -0.72 | -0.05 | -0.66 |
| 95 | -1.02 | -0.46 | -0.56 |
| 96 | -0.06 | 0.21 | -0.27 |
| 97 | -0.42 | -0.25 | -0.17 |
| 98 | 0.20 | -0.05 | 0.25 |
| 99 | -0.03 | 0.09 | -0.12 |
| 100 | 0.52 | 0.17 | 0.35 |
| 101 | -0.84 | -0.27 | -0.57 |
| 102 | -0.62 | -0.85 | 0.23 |
