# Supplementary material for: GRID-independent molecular descriptor analysis and molecular docking studies to mimic the binding hypothesis of γ-aminobutyric acid transporter 1 (GAT1) inhibitors
Source: PeerJ. 2019 Jan 31;7:e6283. doi: 10.7717/peerj.6283 (PMC6360079; doi:10.7717/peerj.6283)
Supplement: Supplemental Information 13 [file peerj-07-6283-s013.docx]

**Supplementary Table 4.** Correlation between the biological activities and top scored vs. selected poses on the basis of ligand-protein interaction profiles of compounds from classes A, B and C.

| **Class** | **Compound** | **-log IC_50_** | **Top-pose Gold score** | **Selected-pose Gold score** |
| --- | --- | --- | --- | --- |
| A | 2 | 1.3098 | 72.22 | 59.86 |
| A | 8 | 0.1871 | 77.7 | 59.4 |
| A | 15 | -0.1761 | 69.22 | 57.68 |
| B | 14 | -0.1461 | 58.04 | 56.73 |
| B | 27 | -0.8388 | 62.13 | 55.49 |
| B | 36 | -1.4771 | 59.45 | 58.13 |
| C | 1 | 1.3979 | 60.46 | 55.55 |
| C | 3 | 0.699 | 49.91 | 49.91 |
| C | 84 | 0.88 | 55.64 | 43.81 |
| C | 37 | -1.57 | 51.489 | 45.53 |
